# Optimization of Sol–Gel Catalysts with Zirconium and Tungsten Additives for Enhanced CF_4_ Decomposition Performance

**DOI:** 10.3390/molecules29215179

**Published:** 2024-11-01

**Authors:** Younghee Jang, Sang Moon Lee, Sung Su Kim, D. Duc Nguyen

**Affiliations:** 1 Graduate School, Kyonggi University, Suwon 16227, Republic of Korea; yhjang39@kyonggi.ac.kr; 2 Department of Civil & Energy System Engineering, Kyonggi University, Suwon 16227, Republic of Korea; catallee@gmail.com

**Keywords:** Ni/ZrO_2_-Al_2_O_3_ catalyst, sol–gel synthesis, zirconium additive, tungsten additive, CF_4_ decomposition

## Abstract

This study investigated the development and optimization of sol–gel synthesized Ni/ZrO_2_-Al_2_O_3_ catalysts, aiming to enhance the decomposition efficiency of CF_4_, a potent greenhouse gas. The research focused on improving catalytic performance at temperatures below 700 °C by incorporating zirconium and tungsten as co-catalysts. Comprehensive characterization techniques including XRD, BET, FTIR, and XPS were employed to elucidate the structural and chemical properties contributing to the catalyst’s activity and durability. Various synthesis ratios, heat treatment temperatures, and co-catalyst addition positions were explored to identify the optimal conditions for CF_4_ decomposition. The catalyst composition with 7.5 wt% ZrO_2_ and 3 wt% WO_3_ on Al_2_O_3_ (3W-S3) achieved over 99% CF_4_ decomposition efficiency at 550 °C. The study revealed that the appropriate incorporation of ZrO_2_ enhanced the specific surface area and prevented sintering, while the addition of tungsten further improved the distribution of active sites. These findings offer valuable insights into the design of more efficient catalysts for environmental applications, particularly in mitigating emissions from semiconductor manufacturing processes.

## 1. Introduction

Perfluorocarbons (PFCs) are prominent greenhouse gases emitted from semiconductor facilities [1], possessing greenhouse gas indices ranging from approximately 1200 to 23,900 times that of carbon dioxide [2,3]. Their presence alone significantly contributes to global warming concerns, with CF_4_ recognized as one of the most tightly bound substances among PFCs [3,4,5]. Various methods including plasma [4,6,7,8,9,10], catalytic hydrolysis [11,12,13,14,15], combustion [2], and adsorption [3,16,17,18,19,20,21,22] have been extensively researched for PFC mitigation [2,4,6,11,12,13,14,15,16,17,23,24]. Due to its extremely strong bond (C-F bond energy is CF_4_ is 543 ± 4 kJ mol) and symmetrical structure, a higher temperature is necessary to crack the CF_4_ molecule [3]. Conversely, catalytic hydrolysis, known for its lower activation energy, as indicated by Equation (1), is regarded as the most promising technology. In addition to its economic feasibility, catalytic hydrolysis reduces energy consumption by operating at lower temperatures. It offers better control over by-products, such as HF, making it an environmentally superior alternative to thermal decomposition [25,26].

CF_4_ + 2H_2_O→CO_2_ + 4HF (1)

While CO_2_ is produced during the decomposition of CF_4_, this method still presents a significant environmental advantage. CF_4_, due to its strong C-F bonds and stability, is much more difficult to break down and poses a greater threat to the environment. The conversion to CO_2_, a less harmful greenhouse gas, allows for a more manageable environmental impact. Additionally, with the advancements in carbon capture, utilization, and storage (CCUS) technologies, CO_2_ can be efficiently sequestered or repurposed in industrial applications. Thus, this process not only mitigates the emissions of a more potent greenhouse gas, but also leverages existing technologies to further reduce the overall carbon-contribution to climate change.

In the 1980s, research focused on weakly bound compounds such as CCl_4_ and CFCl_3_ [19,27,28,29,30], reporting efficiency rates of around 99% at temperatures of approximately 773 K. As we entered the 21st century, there has been a concentrated effort on developing catalysts for CF_4_ decomposition. El-Bahy et al. [14,31] evaluated the effects of various active metals on γ-Al_2_O_3_ supports, reporting over 95% activity at around 973 K with metals such as Zn, Zr, Ga, Cr, and Ni. Thus, CF_4_ decomposition by alumina-based catalysts requires thermal energy above 700 °C. Previous studies [11,13,14,31] on CF_4_ decomposition using rare earth element precursors have shown that the starting temperature for CF_4_ decomposition is around 550 °C. This study suggests that light rare earth promoters such as Nd, Ce, La, and Pr enhance catalyst activity. Song et al. [13] demonstrated that Al_2_O_3_-based catalysts modified with cerium sulfate (Ce(SO_4_)_2_) enabled stable CF_4_ decomposition. They reported that the addition of cerium sulfate precursor, compared to cerium nitrate, resulted in an increased amount of Lewis acid sites on the catalyst, which they identified as the main effect necessary for breaking C-F bonds. The addition of cerium sulfate adjusts the charge density on the catalyst surface and creates oxygen vacancies, which enhances Lewis acid sites by controlling oxygen species. Reddy et al. [32] added zirconium to activate Lewis acid sites and investigated the combination structure of Al^3+^ and Zr^4+^. Luo et al. [33] reported that adding zirconium species through Zr-Si synthesis promotes Lewis acid site formation. Zheng et al. [15] selected zirconium from previous studies and obtained Zr/Al_2_O_3_ catalysts, reporting a CF_4_ decomposition efficiency of 85% at 923 K.

Despite the initial step of CF_4_ decomposition being attributed to Lewis acid sites, the long-term ability of a catalyst to decompose CF_4_ is considered to be governed by other characteristics. Since hydrogen fluoride (HF) is one of the main products that react after CF_4_ decomposition, it is crucial to identify a catalyst that can withstand the acidic conditions caused by HF [34]. HF induces changes in the morphology of the catalyst as a side reaction occurs on the catalyst surface. As shown in Equations (2) and (3), HF forms AlF_3_ around 773 K and transforms into the alpha phase Al_2_O_3_ at above temperature [34,35,36]. Xiu et al. [35,36] reported that the effect of HF on γ-Al_2_O_3_ catalysts is highly temperature-dependent. In CF_4_ hydrolytic decomposition under excess water, γ-Al_2_O_3_ transforms into AlF_3_ below 873 K, while α-Al_2_O_3_ forms above 873 K. α-Al_2_O_3_ has a minimal surface area and low Lewis acidity, such a phase transition is regarded a significant pathway for catalyst deactivation [37,38]. Therefore, it is important to prepare a catalyst resistant to HF is crucial for effective PFC decomposition without deactivation.

Most researchers have attempted to prevent the phase transition using many metals such as Zn [25,31,36], Ga [14,31], Ni [25,31], Zr [15], phosphate [35], and alkaline earth elements [25]. Generally, these additives can enhance the stability of the catalyst by slowing down the phase transition process. However, they frequently lead to a significant decrease in catalytic activity due to the reduction in the number of Lewis acid sites [25].

Recent studies have reported that combining catalyst and plasma technology has successfully enhanced the overall decomposition efficiency. While the plasma-alone system could only reach around 50% efficiency, incorporating γ-Al_2_O_3_ catalysts achieved 100% CF_4_ decomposition. This improvement can be focused on the significant role of γ-Al_2_O_3_ in plasma-catalyst synergy, effectively boosting the CF_4_ decomposition performance [39,40]. Additionally, catalysts using rare earth elements and sulfates have shown significant improvement in CF_4_ decomposition efficiency [41]. Chen et al. [42] reported that Zr-based catalysts modified with sulfate achieved 100% CF_4_ decomposition at 580 °C, a significantly lower temperature compared to conventional catalysts. Structural modifications of Al_2_O_3_-based catalysts to enhance resistance to HF corrosion have also become an essential research direction. θ-Al_2_O_3_, for example, was shown to have 20 times the stability compared to γ-Al_2_O_3_, making it a promising catalyst for long-term CF_4_ decomposition [43].

Furthermore, Wang et al. [44] reported that oxide-based catalysts such as ZnAl_2_O_4_ were effective for CF_4_ decomposition. Various acid treatments (e.g., H_2_SO_4_, HCl, HNO_3_) were used to increase the proportion of Al(III) sites, thereby activating Lewis acid sites and promoting CF_4_ decomposition. H_2_SO_4_-treated ZnAl_2_O_4_ achieved 100% CF_4_ decomposition at 600 °C, demonstrating superior stability compared to previous catalysts [42,44]. These studies aimed to overcome the limitations of conventional CF_4_ decomposition catalysts by developing new catalysts that maintain high efficiency at lower temperatures and under more stable conditions.

Al_2_O_3_ + 6HF→2AlF_3_ + 3H_2_O (2)

2AlF_3_ + 3H_2_O→α-Al_2_O_3_ + 6HF (3)

Considering the aforementioned reasons, this study proposed a granule-type Zr-Al catalyst capable of efficiently decomposing CF_4_ at temperatures below 973 K while maintaining durability. This research aimed to determine the optimal synthesis conditions for the Zr-Al support and investigate the role of tungsten as a co-catalyst in enhancing activity. The physicochemical properties of the catalyst were confirmed through XRD, BET, and FTIR analyses. Each catalyst was evaluated based on the analytical results and CF_4_ decomposition performance to determine the optimal ratio for enhancing activity.

## 2. Results and Discussion

### 2.1. Characterization of the Ni/ZrO_2_-Al_2_O_3_

**Figure 1:**
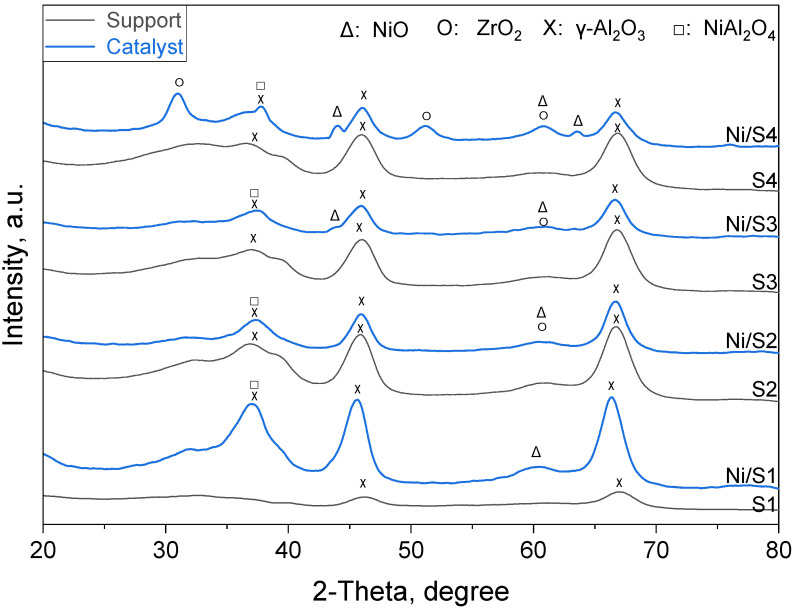
XRD patterns of supports and catalysts with various zirconium contents.

Zirconia (ZrO_2_) is a thermally stable ceramic material that prevents particle fusion within metal and Al_2_O_3_ at high temperatures. They can prevent the sintering of the active metal as well as ensure a high specific surface area [45,46,47]. Zheng et al. [15] reported that Zr-modified γ-Al_2_O_3_ catalysts achieved a higher CF_4_ conversion rate (85% at 650 °C) and maintained stability for over 60 h compared to unmodified γ-Al_2_O_3_, which showed a much lower conversion rate and faster deactivation due to sintering. The inclusion of zirconium also helps by interacting with nickel or other active metal particles, reducing their aggregation at high temperatures, which is a common cause of sintering [48]. Liu et al. [49] incorporated ZrO_2_ to inhibit the sintering of nickel particles during high-temperature reactions. In this study, boehmite and zirconium oxynitrate were reacted using the sol–gel method to manufacture the support. As a result, an abrupt decrease in pH and an acceleration in gelation rate were observed with increasing zirconium content. XRD analysis was performed to verify the surface morphology and crystallinity of the prepared support and catalyst (Figure 1). The Al_2_O_3_ particles (JCPDS file No. 50-0741) showed that the diffraction characteristic peaks at 2θ were 32.1°, 37.6°, 39.7°, 45.8°, 66.8°, which corresponded to the planes (220), (311), (222), (400), and (440) [50,51]. The ZrO_2_ (JCPDS No. 79-169) peaks were observed at 30.2°, 35.0°, 50.4°, 60.0°, 62.8°, and 74.0°, which linked to planes (111), (200), (220), (311), (222), (400), (331), and (420), respectively [52,53]. Sample S4 distinctly showed ZrO_2_ formation after high-temperature treatment. The NiO XRD pattern (JCPDS No. 47-1049) revealed diffraction peaks at 2θ of 37.7°, 43.8°, and 63.3°, associated with the planes (111), (200), and (220), respectively [51]. Moreover, no NiO peaks were observed, indicating effective nickel dispersion on the gamma Al_2_O_3_, minimizing the formation of large NiO species. Significant differences were observed (Table 1). S1, without zirconium, had a surface area of 218.09 m^2^∙g^−1^, which decreased to 15.90 m^2^∙g^−1^ after nickel addition, likely due to blocking NiO in the pores of Al_2_O_3_. In contrast, S2, S3, and S4, which contained zirconium, showed a smaller reduction in surface area. This suggests that zirconium plays a role in preventing both blocking and sintering. S2 and S3 had surface area reductions of less than 2%, while S4 showed a 25% decrease. S4 demonstrated strong resistance to sintering compared to Al_2_O_3_, which lost over 90% of its surface area. Similarly, Liu et al. [49] reported a loss of over 85% in specific surface area for Ni/Al_2_O_3_ catalysts during high-temperature reactions. Incorporating ZrO_2_ reduced the surface area loss and enhanced thermal stability, attributed to the partial coverage of Ni particles by ZrO_2_, preventing particle growth and strengthening the interaction between Ni and the support under reaction conditions.

These results suggest that an appropriate ZrO_2_ content (in this study, 5 wt.% to 7.5 wt.%) that does not form surface ZrO_2_ has a crucial role in preventing Al_2_O_3_ sintering, thus maintaining a high BET surface area and stable support structure without increasing crystalline intensity. Heat treatment temperature is a key factor in controlling the catalyst structure. Figure S1 shows the XRD patterns of supports and catalysts prepared at various heat treatment temperatures (673 K, 773 K, 873 K, 973 K), with nickel loading followed by heat treatment at 973 K. As shown in Figure S1, higher heat treatment temperatures led to more pronounced ZrO_2_ peaks, indicating ZrO_2_ coagulation.

When the support was heat-treated at a lower temperature (573 K), the zirconia and alumina precursors did not bond effectively, causing isolated ZrO_2_ to appear on the surface after the final 973 K treatment. Similarly, even at higher temperatures (873 K, 973 K), if the support was not properly stabilized, ZrO_2_ still surfaced. This emphasizes the importance of optimal heat treatment for producing a stable and active catalyst. Additionally, Li et al. [54] found that increasing the annealing temperature improved the interfacial bonding between graphene and alumina. Liu et al. [55] reported that insufficient heat treatment left residual precursors, causing rapid ZrO_2_ coalescence during high-temperature treatment due to leaving residual zirconium precursors under inadequate heat treatment. At 773 K, only the nitrate precursor bonds are broken, maintaining a state just before ZrO_2_ and Al_2_O_3_ bonding. Sufficient thermal energy broke the precursor bonds by 873 K and 973 K, allowing for increased surface crystallinity as observed through XRD, enhancing surface crystalline growth.

**Figure 2:**
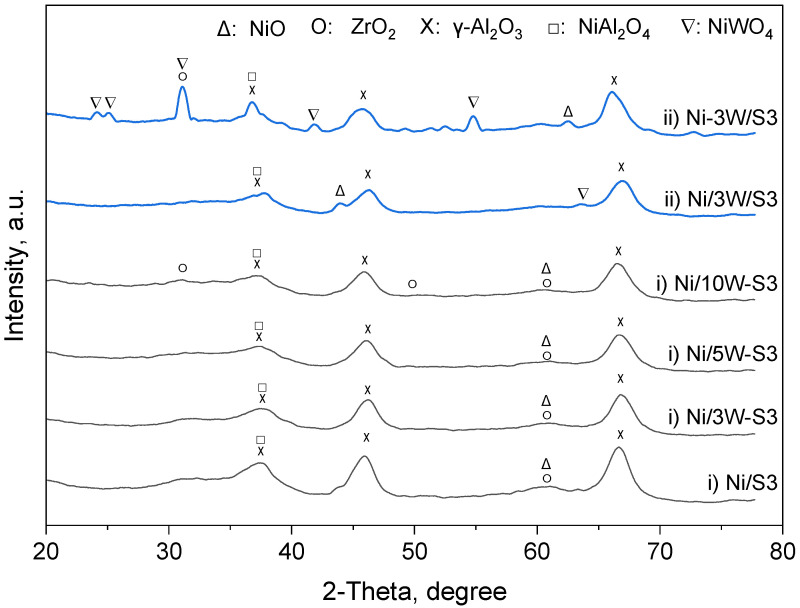
XRD patterns of catalysts based on the tungsten addition i) amount and ii) position.

Figure 2 shows the XRD results of the catalysts with varying amounts of tungsten (W) addition. Similar to Figure 1, the appropriate addition of W within the stable synthesis of 3S showed no structural changes in the support. However, in the case of Ni/10W-3S with 10% W addition, ZrO_2_ was observed at 30.2°. This was attributed to the excessive addition of W interfering with the bonding between ZrO_2_ and Al_2_O_3_. Nevertheless, the W content did not affect the specific surface area. The Ni/S3 catalyst exhibited a specific surface area of 195.14 m^2^·g^−1^, while Ni/3W-S3, Ni/5W-S3, and Ni/10W-S3 maintained specific surface areas of 165.38 m^2^·g^−1^, 166.12 m^2^·g^−1^, and 169.74 m^2^·g^−1^, respectively.

As shown in Figure 2(ii), the addition of tungsten (W) at specific positions induced changes in the catalyst structure. In the Ni-W/S3 catalyst, peaks corresponding to NiWO_4_ [56] were observed at 23.89°, 24.85°, 30.87°, 31.5°, 36.6°, 39.1°, 41.7°, 44.7°, 46.4°, 49°, 51.1°, 52.3°, 53.2°, 54.6°, 62.3°, 65.5°, 68.9°, and 72.6°, indicating reactions between Ni and W. The formation of NiWO_4_ was identified through these diffraction patterns, which corresponded to the reference data (JCPDS No. 15-0755). During catalyst preparation, heat treatment at temperatures higher than the reaction conditions ensured structural stability, facilitating the formation of NiWO_4_, which is crucial for improved catalytic performance. In the Ni/W/S3 catalyst, peaks corresponding to Al_2_O_3_ [57] were observed at 32.7°, 36.8°, 39.5°, 45.4°, and 67.2°. Although maintaining a structure similar to the initially synthesized catalyst, increased heat treatment led to sintering phenomena and decreased the specific surface area, consistent with previous research findings.

### 2.2. Acid-Base Properties of the Ni/ZrO_2_-Al_2_O_3_

**Figure 3:**
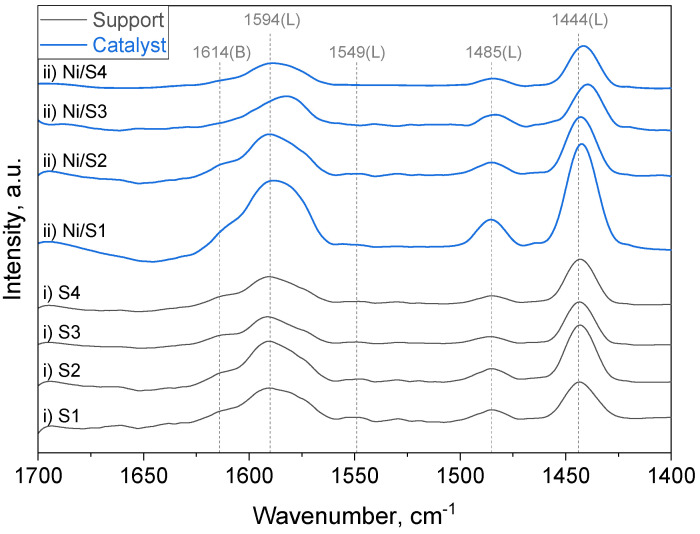
Infrared spectroscopy of pyridine adsorption (Py−IR) on i) supports or ii) catalysts prepared with various zirconium contents at room temperature.

**Figure 4:**
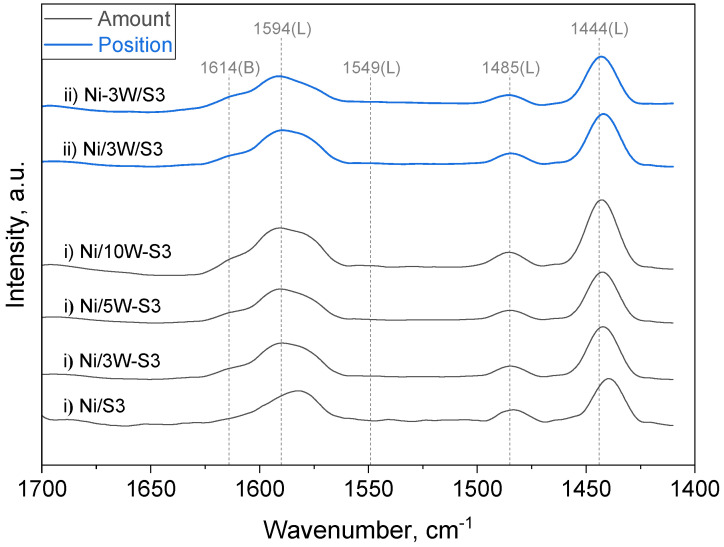
Infrared spectroscopy of pyridine adsorption (Py−IR) profiles of catalysts based on tungsten addition i) amount and ii) position.

Figure 3 and Figure 4 depict the scatter plots of supports and catalysts using pyridine adsorption FTIR. CF_4_ decomposition reactions initiate at Lewis acid sites, making it essential to identify and quantify the amount of Lewis acid sites in the catalysts using this method. Spectra were found over a frequency range of 1400 cm^−1^ to 1700 cm^−1^, where characteristic vibration modes of adsorbed pyridine were observed [15,33,58]. These peaks at 1445 cm^−1^, 1486 cm^−1^, 1588 cm^−1^, and 1613 cm^−1^ indicate Lewis acid sites, while peaks at 1530 cm^−1^, 1550 cm^−1^, and 1570 cm^−1^ indicate Brønsted acid sites [55]. As a result, the scatter plots of the support surface predominantly showed Lewis acid sites at room temperature, with increased intensity observed upon the addition of ZrO_2_. In contrast, the pyridine adsorption FTIR of the catalyst indicated strong intensity for Ni/S1, as shown by the blue line.

Nickel plays an important role in enhancing both reduction and oxidation reactions, especially under high-temperature conditions. As shown Figure 3, nickel can strengthen the Lewis acid sites on the support, as seen in the increased intensity in Figure 3. Ni/S1 showed significant Lewis acid site enhancement, which correlates with role in improving stable active sites for catalytic processes. However, despite the strong activity observed for Ni/Al_2_O_3_, no significant variation was observed in relation to ZrO_2_ content, suggesting that the primary role of ZrO_2_ is to stabilize the catalyst and prevent sintering, rather than further enhancing the Lewis acidity.

Ni et al. [59] synthesized Ni/ZrO_2_ using various methods (incipient-wetness impregnation (iwi), positive co-precipitation (pc), and parallel flow co-precipitation (pfc) methods) for hydrogenation reactions and observed differences in activity. The variation in activity was attributed to the electron density and distribution of nickel, which was found to be correlated with the oxygen deficiency of the ZrO_2_ support.

Interestingly, tungsten oxides are well-known for their reducible nature, allowing control over the electron density of the active metal and maintaining numerous oxygen vacancies within the support lattice. The characteristics of various tungsten oxides suggest significant potential for CF_4_ decomposition. Indeed, numerous studies [32,60,61,62,63,64,65] have reported that tungsten oxides play a role in introducing acidic sites in most catalysts. Particularly, Lewis acidic sites are generated by unsaturated W^6+^ species, while Brønsted acidic sites are associated with hydroxyl groups formed by bridging Si-O-W or terminal W=O bonds [60,64,66,67]. Figure 4 also demonstrates that the addition of tungsten enhanced the acidity of the catalyst. As the loading of W increased, the intensity also increased. Furthermore, by varying the position of the loading without observing structural sintering, the catalyst with 3W loading in S3 showed the highest acidity. In other words, Lewis acid sites are considered the rate-determining step for CF_4_ decomposition, and catalysts with higher acid strength are expected to exhibit superior initial activity.

**Figure 5:**
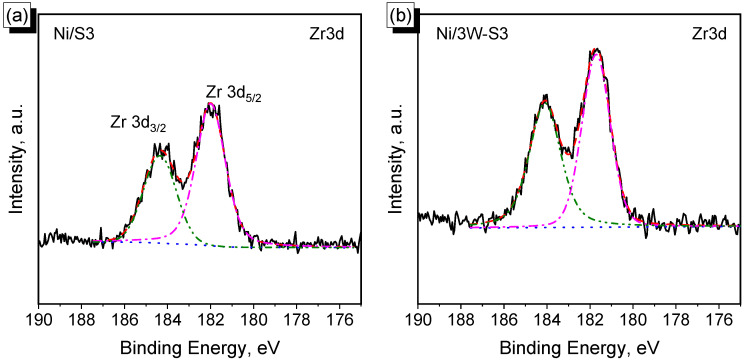
Zr 3d XPS spectra (dash line, pink for Zr 3d5/2 and green for Zr 3d3/2. Dotted line, blue for background, solid line, black for Experimental curve) of the catalysts (**a**) Ni/S3 and (**b**) Ni/3W-S3.

To gain insights into the surface chemical composition of the catalyst, X-ray photoelectron spectroscopy (XPS) analysis was performed, and the results are presented in Figure S2 (Ni2p and O1s) and Figure 5 (Zr3d). As shown in Figure S2a, Ni^2+^ was fully characterized through the Ni2p emission spectrum [68,69]. Peaks at 855.1 eV and 872.8 eV corresponded to Ni^2+^. Figure S2b elucidates the contribution of oxygen. The XPS results of O1s [69] showed a single peak with a shoulder for all samples annealed at different temperatures, with binding energies of 530 eV and 531.5 eV representing oxygen bonds with metals (O-M) and hydroxides (O-OH), respectively. The fitting peak at 530 eV for O1 was attributed to typical metal-oxygen bonds. The peak at 531.5 eV for O_2_ is generally considered a low-oxygen site and surface-adsorbed oxygen species.

As shown in Figure 5, the analysis of the Zr 3d [70,71] showed two main binding energy values located at 182.0 eV, 184.5 eV for Ni/3S, and at 181.7 eV, 184.0 eV for Ni/3W-S3. The binding energies of Ni/3W-S3 were lower than those of Ni/S3, indicating that the W-O bonding environment withdraws more electron density, leading to a more positive charge on the Zr sites. This is expected to increase the quantity and strength of Lewis acid sites on the catalyst, which agrees with the results of the FTIR.

### 2.3. Catalytic Properties of the Ni/ZrO_2_-Al_2_O_3_

**Figure 6:**
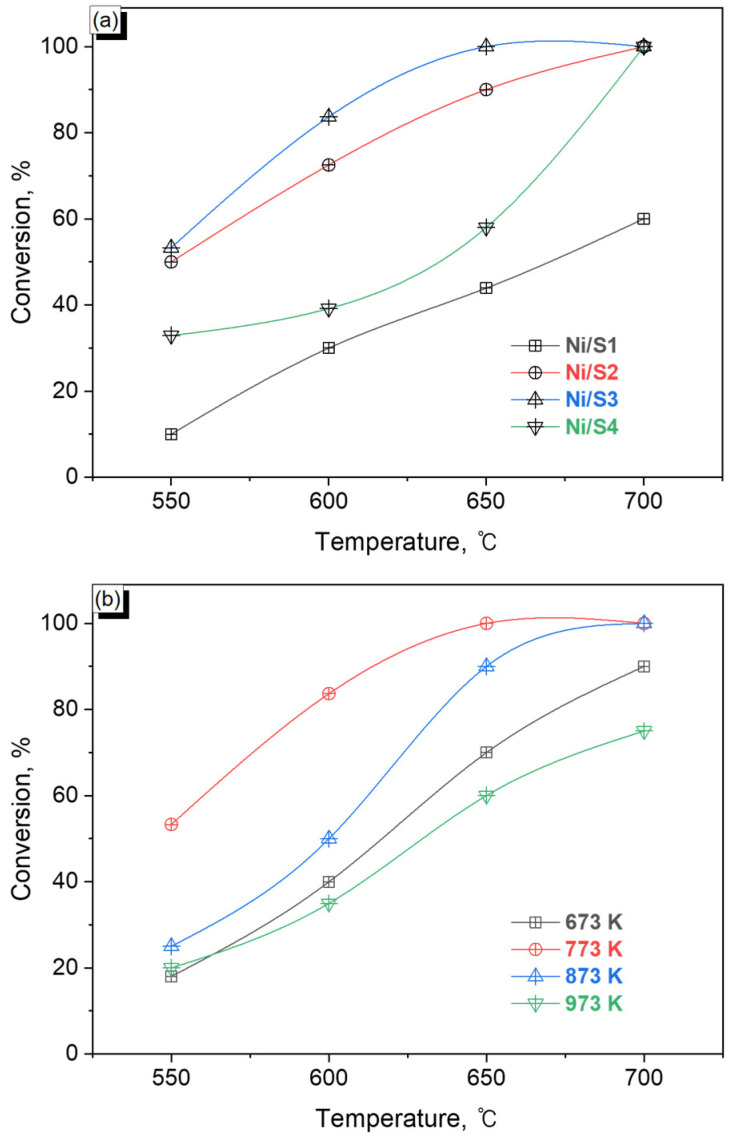
CF_4_ conversion of catalysts with (**a**) various ZrO_2_ contents and (**b**) various calcined temperatures (SV: 4000 h^−1^, 300 cc/min flow, CF_4_: 1000 ppm, H_2_O: 8 *v*/*v*%).

The characteristics of the prepared catalysts can be summarized as follows: the addition of zirconium prevented the sintering of Al_2_O_3_, thereby maintaining its specific surface area, and the acidity increased with the increase in zirconium content. Additionally, variations in the addition ratio of zirconium and tungsten led to differences in the adsorption of CF_4_ and H_2_O. While many researchers [11,12,13,15,25,28,31,34,35] have focused solely on the acidity for CF_4_ decomposition, the contact ratio between CF_4_ and H_2_O was also deemed significant. In this study, the activity of the prepared catalysts was directly confirmed by simultaneously injecting CF_4_ and H_2_O to assess their decomposition efficiency (Figure 6).

Technically, as shown in Figure S3, the initial activity was calculated by determining the conversion rate after approximately 3 h of reaction upon reaching the target temperature. The effect of ZrO_2_ content is illustrated in Figure 6a. The results indicate that the conversion performance was superior in the order of 7.5% > 3% > 15% > 0% ZrO_2_ content, demonstrating that excellent acidity and the adsorption capacity of reactants could maintain the efficiency of CF_4_ decomposition. The CF_4_ decomposition activity of the catalysts at different support calcination temperatures is illustrated in Figure 6b. It is evident that the calcination conditions significantly influenced the CF_4_ decomposition performance. Particularly for Ni/S3 (773 K), approximately 78% activity was observed at 873 K, while for Ni/S3 (873 K), the activity dropped to around 50%. Ni/S3 (673 K) exhibited 38% activity, and even at 973 K, only approximately 90% activity was observed. The catalyst with the least activity was Ni/S3 (973 K) treated at 973 K.

Consistent with the XRD results in Figure S1, all catalysts with grown ZrO_2_ showed reduced activity. This observation is closely related to the structural and thermal stability of the catalysts, which is significantly affected by the calcination temperatures. The key factor determining the performance is the ability to maintain a stable Al_2_O_3_ structure without excessive ZrO_2_ growth. As shown in Figure S1, for supports treated at temperatures other than 773 K, ZrO_2_ was distinctly observed on the surface after catalyst loading, while Ni/S3 (773 K) maintained a relatively stable Al_2_O_3_ structure. This indicates that 773 K is the optimal temperature to achieve a high surface area and effective NiO dispersion, providing the necessary thermal energy for stable ZrO_2_–Al_2_O_3_ interactions. Lower calcination temperatures, such as 673 K, result in insufficient thermal energy, preventing stable ZrO_2_-Al_2_O_3_ bond formation and leading to sintering during subsequent high-temperature exposure. In contrast, higher temperatures like 873 K and above, while enabling ZrO_2_-Al_2_O_3_ bond formation, also induce excessive sintering due to the overaccumulation of thermal energy, which compromises the catalytic activity. Therefore, maintaining a stable structure and preventing ZrO_2_ growth is critical for enhancing CF_4_ decomposition efficiency. This study identified 773 K as the optimal calcination temperature, balancing the need for high specific surface area, stability, and proper dispersion. Similar studies have emphasized that the thermal treatment must be carefully controlled to manage sintering and ensure the optimal dispersion properties [72,73,74,75].

**Figure 7:**
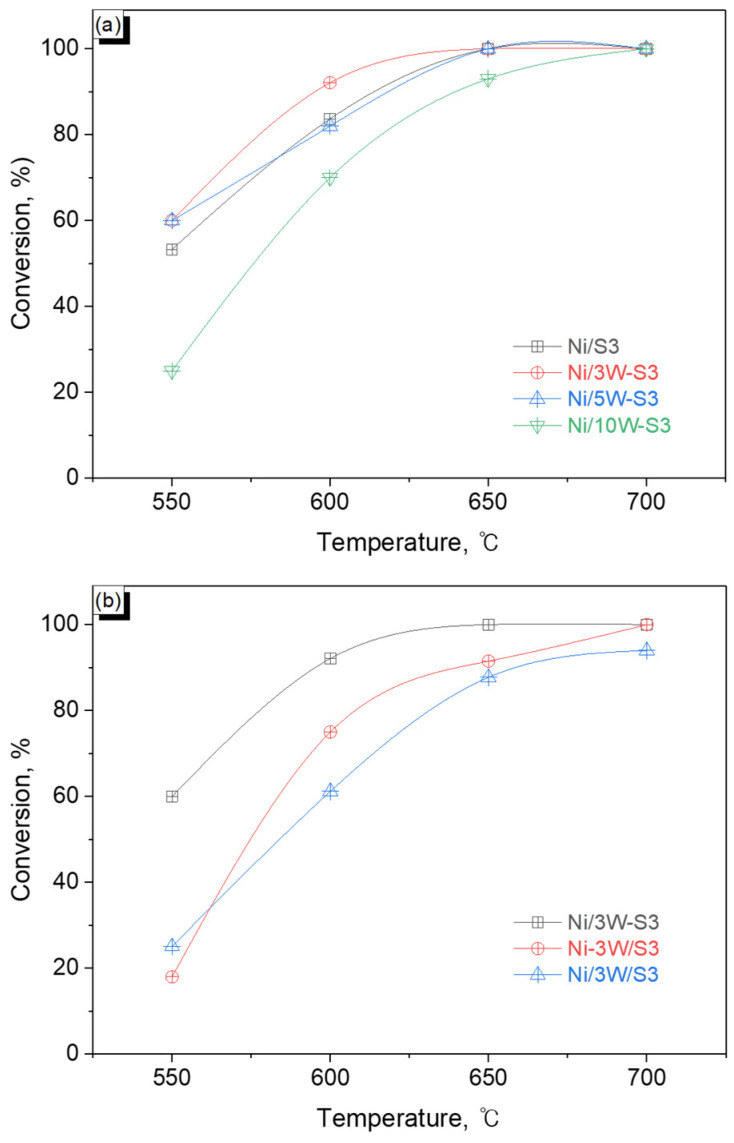
CF_4_ conversion of catalysts with tungsten: (**a**) loadings; (**b**) added position (SV: 4000 h^−1^, 300 cc/min flow, CF_4_: 1000 ppm, H_2_O: 8 *v*/*v*%).

As shown in Figure 2, tungsten (W) was added during the vigorous stirring in the support manufacturing stage, and it was determined that various catalyst structural changes could occur depending on the method of W addition. Catalysts prepared according to the location of W addition exhibited structural differences, as observed in XRD (Figure 2) and BET (Table 1), and the differences in catalytic activity were compared accordingly (Figure 7b). The results indicated that the location of W addition showed the best specific surface area and nickel dispersion when incorporated into the support synthesis process. In the temperature range of 873 K, Ni-3W/S3, which formed NiWO_4_, exhibited a conversion rate of 75%, while Ni/3W/S3 with a reduced specific surface area due to multi-step calcination showed a conversion rate of 61%. This resulted in a decrease in activity of 15% to 30% in the same acid strength range. These findings demonstrate that the structure, specific surface area, and acid strength of the prepared catalysts collectively influence their activity.

**Figure 8:**
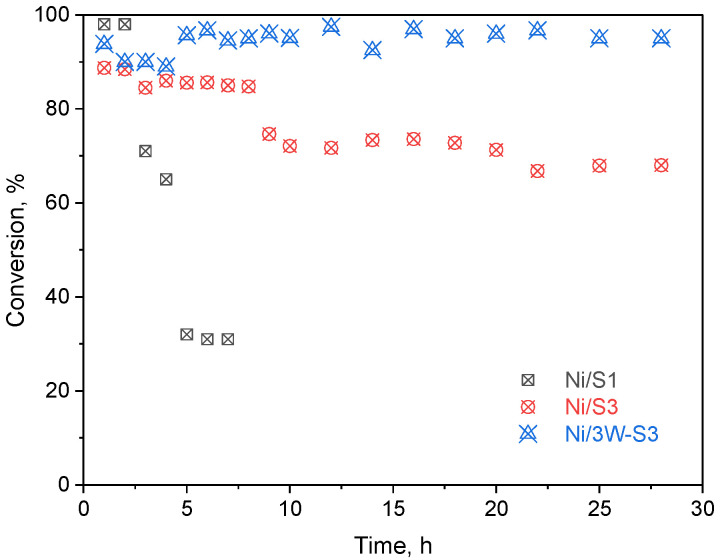
CF_4_ conversion over time for Ni/S1, Ni/S3, and Ni/3W-S3 (SV: 4000 h^−1^, 300 cc/min flow, CF_4_: 1000 ppm, H_2_O: 8 *v*/*v*%).

Figure 8 presents the long-term activity evolution of Ni/S1, Ni/S3, and Ni/3W-S3. Ni/S3 and Ni/3W-S3 with added zirconium exhibited durability for over 30 h, ultimately achieving conversion rates of 68% and 95%, respectively. In contrast, Ni/S1 showed a rapid decline in activity after 3 h, recording a conversion rate of approximately 37% after about 5 h. These results indicate that the developed synthetic supports can maintain sintering prevention and CF_4_ activity compared to conventional Al_2_O_3_, and the addition of W in S3 further enhanced the acidity, thereby strengthening the activity.

**Scheme 1:**
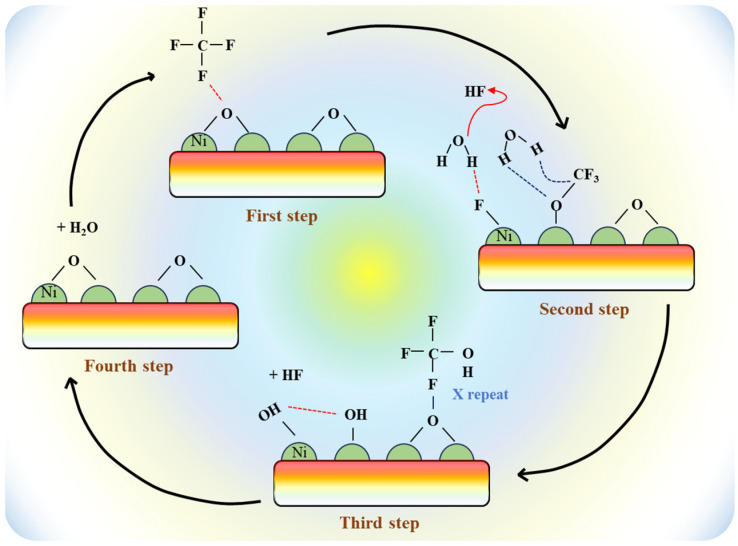
Catalytic reaction mechanism on CF_4_ decomposition (solid, black for acid site; dot, black for adsorbed vapor; red for adsorbed CF_4_).

Scheme 1 is a simplified illustration of the mechanism in which CF_4_ and H_2_O repeatedly adsorb and desorb at Lewis acid sites, reacting to form CO_2_ and HF. Initially, CF_4_ is decomposed into Ni-F and O-CF_3_ upon binding to the Lewis acid sites on the nickel surface, which acts as the active metal. At this point, two moles of H_2_O approach and interact, with hydrogen ions bonding and converting into one mole of HF and OH-CF_3_. After HF is released, Ni-OH groups form on the surface of the active metal. When two Ni-OH groups meet, one mole of H_2_O is released, and the Lewis acid sites are restored. This sequence repeats, resulting in the complete decomposition of one mole of CF_4_, releasing four moles of HF. From the FTIR results, it is clear that a higher absolute adsorption amount of CF_4_ leads to more CF_4_ available for reaction. Furthermore, it can be understood that the overly rapid desorption of CF_4_ has a negative impact, as it prevents sufficient decomposition reactions from occurring on the surface of the active metal. Various catalyst preparation methods were employed to enhance performance, and the addition of ZrO_2_ and the co-catalyst W further optimized the catalytic activity.

## 3. Experimental Setup

### 3.1. Catalyst Preparation and Characterization

#### 3.1.1. Preparation of Support

The catalyst employed nickel as the active metal, while boehmite (CATAL A, Sasol Co., Johannesburg, South Africa) served as the support. The catalyst was prepared through a two-step process using sol–gel and wet impregnation methods. Metal oxides used in support synthesis were mixed with various proportions of zirconium(IV) oxynitrate (ZrO(NO_3_)_2_) and boehmite (AlO(OH)), with the nickel content impregnated into the support calculated at 10% by weight of the catalyst. After the sol–gel reaction was completed, the mixture was dried completely in an oven at 378 K. The dried support was sieved to a particle size of 12–25 mesh and then calcined at temperatures between 373 K and 973 K. The active metal was dissolved in distilled water, mixed with the support, and then coated by vacuum evaporation at 338 K. The catalyst was finally obtained by calcining at 973 K for 2 h. The prepared supports were named according to Table 2 and were denoted as Ni/support for catalysts coated with active metals. For example, if the 3W-S3 support was used, it would be indicated as Ni/3W-S3.

#### 3.1.2. Activity Tests

Catalyst activity was performed in a reaction system consisting of a gas flow, a catalyst reactor, an HF removal scrubber, and a GC instrument for the analysis of unreacted CF_4_. The experimental gas concentrations included CF_4_ at 1000 ppm and H_2_O at 8 *v*/*v*%. To minimize the effect on the catalyst’s uniformity and pressure loss, the prepared catalyst was crushed and sieved to a 12–25 mesh size. The reactor temperature was controlled using a K-type thermocouple located at the top of the fixed-bed (PID Controller, Nova Co., Precision Digital Corporation, Hopkinton, MA, USA). For the reaction part, after reaching the target temperature, the conversion rate after approximately 3 h of reaction was calculated as the initial activity.

The HF removal system serves to prevent the leakage of corrosive acid, HF, from the generated gas after the reaction is complete, both within the analyzer and into the atmosphere. For the analysis part, concentrations of CF_4_ were measured with a GC-TCD (Agilent 6890A, Porapak Q column, Supelco Inc. Corporate, Bellefonte, PA, USA). The catalytic performance in this research was calculated by CF_4_ conversion with Equation (3) and unreacted CF_4_ concentration.

#### 3.1.3. Characterization

The crystal growth and the structure of the material of prepared material were examined by XRD (X-ray diffraction pattern, D/MAX-2200 Ultima, Rigaku, Tokyo, Japan) analysis. Additionally, the obtained XRD patterns were analyzed using Rietveld refinement, calculated with the Rietan-2000 program provided by Rigaku. A BET analysis was performed to compare the pore changes resulting from the mixing ratio and heat treatment of the catalyst. For in situ FTIR analysis, a NICOLET iS10 (Thermo Scientific Co., Waltham, MA, USA) was utilized. Single-beam spectra of thoroughly dried samples at 110 °C were measured as background for collecting the catalyst spectra, and all analyses were conducted with an auto-scan at a resolution of 4 cm^−1^. X-ray photoelectron spectroscopy (XPS) was also performed using an ESCALAB 250Xi (Thermo Scientific) to analyze the valence states of Ni, O, and Zr. The XPS data were calibrated using the C 1s peak at 284.6 eV to ensure accuracy.

## 4. Conclusions

The preparation of monolith-type catalysts via the sol–gel method for CF_4_ decomposition provided several key insights:

- The investigation revealed that introducing zirconium significantly improved the catalyst’s specific surface area and sintering resistance, which are crucial for maintaining high catalytic activity. The addition of tungsten further optimized the distribution of active sites, contributing to the superior performance of the catalyst. However, excessive amounts or incorrect placement of these additives led to structural changes that negatively impacted the catalyst activity.
- The analysis of the catalyst surface exposed to CF_4_ revealed that while the addition of tungsten increased the surface acidity, excessive acidity led to a reduction in CF_4_ adsorption capacity, thereby lowering the reaction rate. When NiO species formed strong bonds on the surface, a decline in catalytic activity was observed, indicating the need for an optimal tungsten content to balance acidity and maintain high catalytic performance.
- Long-term exposure tests revealed that conventional Al_2_O_3_ catalysts demonstrated excellent initial activity, which rapidly declined after 3 h. In contrast, the sol–gel-prepared catalysts exhibited superior durability and maintained both activity and stability, despite having lower acidity compared to Ni/S1. This underscores the critical role that surface acidity plays in both initial catalytic activity and long-term durability. The addition of zirconium further enhanced durability by preventing sintering at high temperatures, while optimizing the tungsten content improved the surface acidity, leading to a significant enhancement in initial activity. These findings suggest that balancing acidity and surface area through appropriate material modifications is key to achieving high performance in catalytic applications.

## Figures and Tables

**Table 1:** Physical properties of the various supports and catalysts.

Catalysts	The Crystal Size of Al_2_O_3_, nm	BET Surface Area, m^2^∙g^−1^
S1	38.6	218.09
S2	42.5	224.56
S3	32.5	197.73
S4	39.5	183.21
Ni/S1	52.5	15.90
Ni/S2	48.2	224.08
Ni/S3	45.9	195.14
Ni/S4	32.5	140.34
Ni/3W-S3	54.7	165.38
Ni/5W-S3	40.0	166.12
Ni/10W-S3	50.8	169.74
Ni-3W/S3	50.5	167.02
Ni/3W/S3	61.6	146.70

**Table 2:** Elemental content analysis of the prepared supports.

Samples	Elemental Composition of Supports		
	Zirconium	Tungsten	Boehmite
S1	0	0	100
S2	3	0	97
S3	7.5	0	92.5
S4	15	0	85
3W-S3	7.5	3.0	89.5
5W-S3	7.5	5.0	87.5
10W-S3	7.5	10.0	82.5

## Data Availability

The authors confirm that the data supporting the findings of this study are available in the paper. Raw data supporting the findings of this study are available from the corresponding author upon request.

## Supplementary Materials

The following supporting information can be downloaded at: https://www.mdpi.com/article/10.3390/molecules29215179/s1, Figure S1: XRD patterns of S3 and Ni/S3 with various calcined temperatures; Figure S2: Ni 2p (a) and O 1s (b) XPS spectra of the catalysts; Figure S3: CF_4_ conversion over time for catalysts prepared with various ZrO_2_ contents (a) 973 K and (b) 923 K; Figure S4: FT−IR spectra of (a) CF_4_ adsorbed and (b) desorbed on catalysts as various ZrO_2_ contents; Figure S5: FT−IR spectra of (a) CF_4_ adsorbed and (b) desorbed on catalysts based on tungsten addition amounts.
