# Supplementary material for: Optimization of Sol–Gel Catalysts with Zirconium and Tungsten Additives for Enhanced CF4 Decomposition Performance
Source: Molecules. 2024 Nov 1;29(21):5179. doi: 10.3390/molecules29215179 (PMC11547982; doi:10.3390/molecules29215179)
Supplement: Supplementary file 1 [file molecules-29-05179-s001.zip › molecules-3196872-supplementary.pdf]

Supplementary data

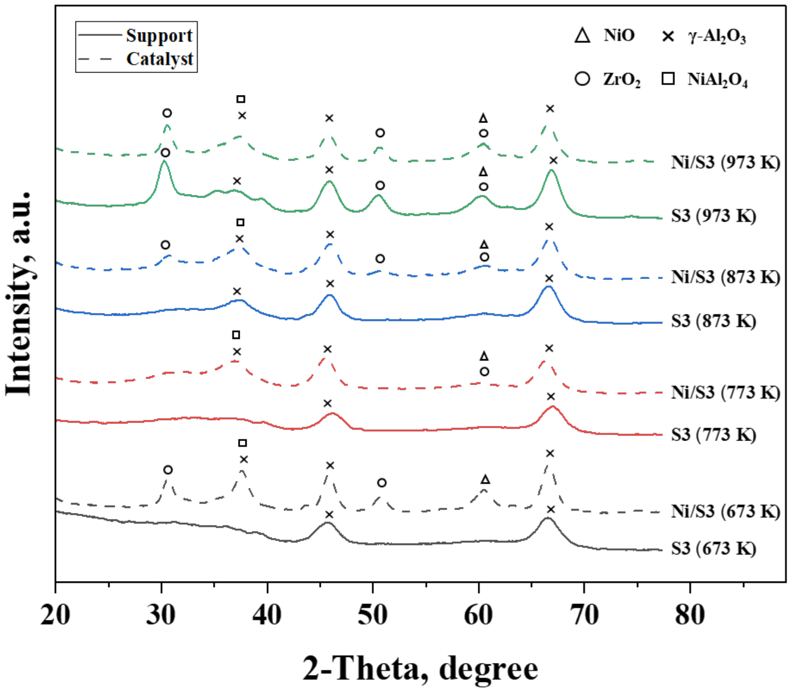

Figure S1. XRD patterns of S3 and Ni/S3 with various calcined temperatures.

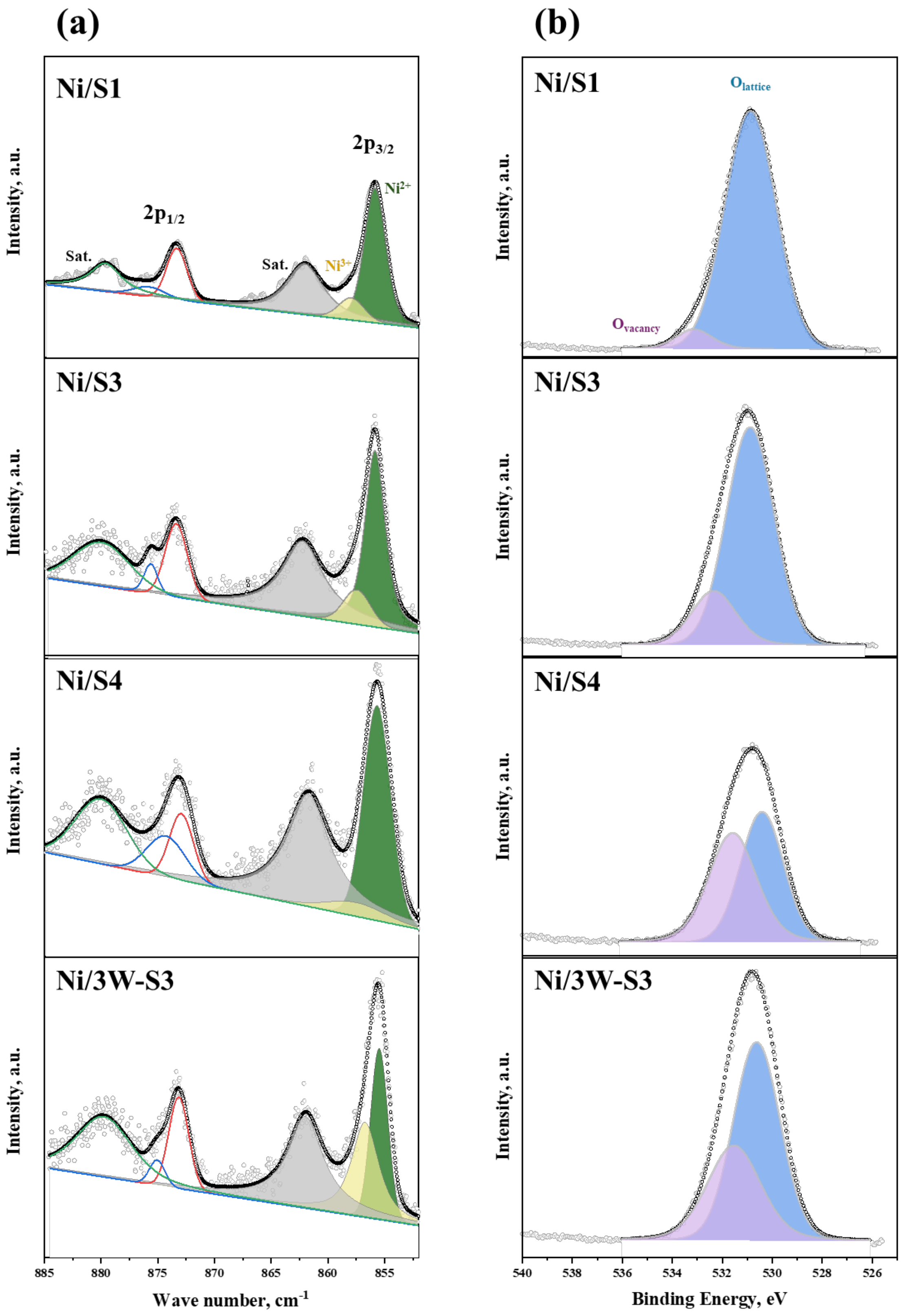

Figure S2. Ni 2p(a) and O 1s(b) XPS spectra of the catalysts.

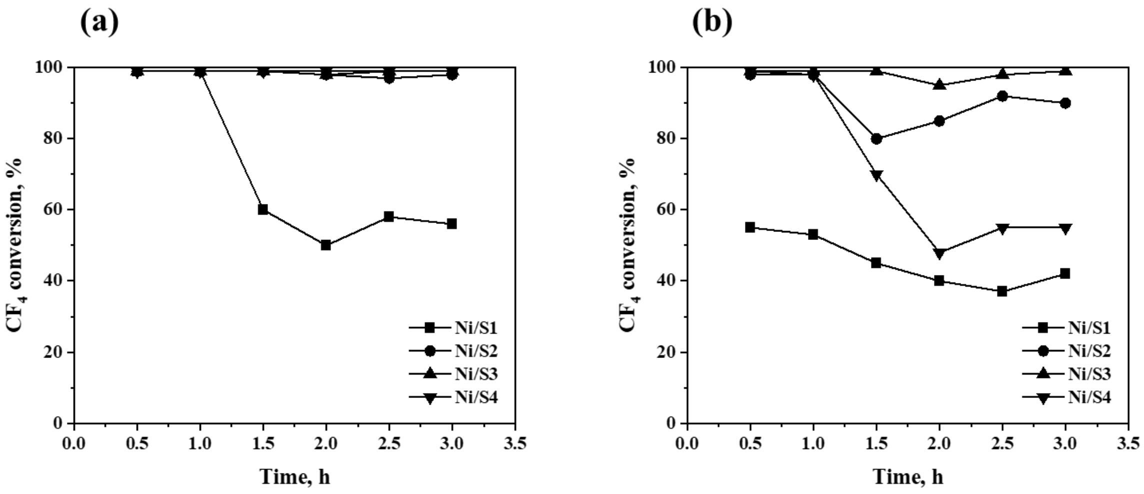

Figure S3. CF_4 conversion over time for catalysts prepared with various ZrO_2 contents (a) 973 K and (b) 923 K.

(SV: 4,000 h^{-1}, 300 cc/min flow, CF_4: 1000ppm, H_2O: 8 v/v%)

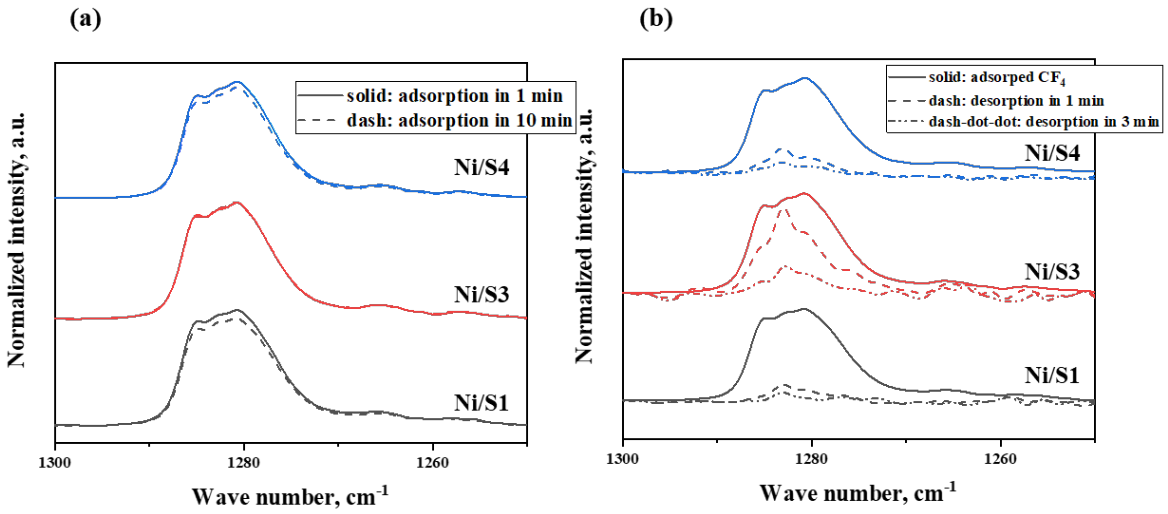

Figure S4. FT−IR spectra of (a) CF_4 adsorbed and (b) desorbed on catalysts as various ZrO_2 contents.

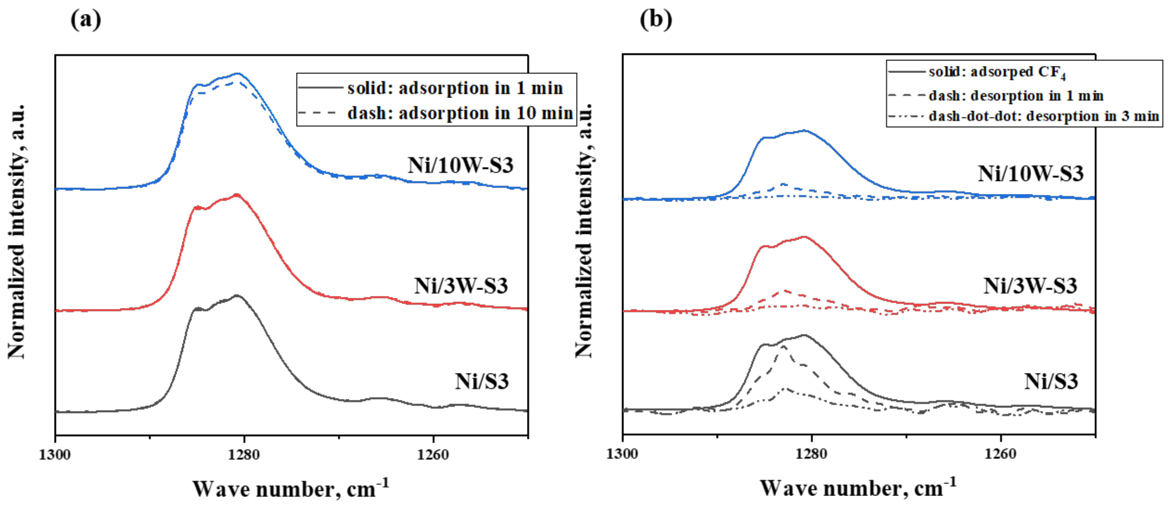

Figure S5. FT−IR spectra of (a) CF_4 adsorbed and (b) desorbed on catalysts based on tungsten addition amounts. Solid line
